# Risk factors of SARS-CoV-2 infection in cancer patients pre- and post-vaccination

**DOI:** 10.1371/journal.pone.0272869

**Published:** 2022-08-09

**Authors:** Suliman A. Alsagaby, Naif Khalaf Alharbi, Fahad A. Alhumaydhi, Faisal Alsubaie, Mohammad Bosaeed, Abdulrhman Aljouie, Abdullah M. Assiri, Kanan Alshammari

**Affiliations:** 1 Department of Medical Laboratories Sciences, College of Applied Medical Sciences, Majmaah University, AL-Majmaah, Saudi Arabia; 2 King Abdullah International Medical Research Center (KAIMRC), Riyadh, Saudi Arabia; 3 King Saud bin Abdulaziz University for Health Science (KSAU-HS), Riyadh, Saudi Arabia; 4 Department of Medical Laboratories, College of Applied Medical Sciences, Qassim University, Buraydah, Saudi Arabia; 5 Assistant Agency for Preventive Health, Ministry of Health, Riyadh, Saudi Arabia; 6 King Abdulaziz Medical City (KAMC), Ministry of National Guard–Health Affairs (MNG-HA), Riyadh, Saudi Arabia; University of Hail, SAUDI ARABIA

## Abstract

**Background:**

Severe complications from COVID-19 and poor responses to SARS-CoV-2 vaccination were commonly reported in cancer patients compared to those without cancer. Therefore, the identification of predisposing factors to SARS-CoV-2 infection in cancer patients would assist in the prevention of COVID-19 and improve vaccination strategies. The literature lacks reports on this topic from the Kingdom of Saudi Arabia (KSA). Therefore, we studied clinical and laboratory data of 139 cancer patients from King Abdulaziz Medical City, Riyadh, KSA.

**Methods:**

The cancer patients fall into three categories; (i) uninfected with SARS-CoV-2 pre-vaccination and remained uninfected post-vaccination (control group; n = 114; 81%), (ii) pre-vaccination infected group (n = 16; 11%), or (iii) post-vaccination infected group (n = 9; 6%). Next, the clinical and lab data of the three groups of patients were investigated.

**Results:**

Comorbidity factors like diabetes and hemodialysis were associated with the risk of infection in cancer patients before the vaccination (*p*<0.05). In contrast to breast cancer, papillary thyroid cancer was more prevalent in the infected patients pre- and post-vaccination (*p*<0.05). Pre-vaccination infected group had earlier cancer stages compared with the control group (*p* = 0.01). On the other hand, combined therapy was less commonly administrated to the infected groups versus the control group (*p*<0.05). Neutrophil to lymphocyte ratio was lower in the post-vaccination infected group compared to the control group (*p* = 0.01).

**Conclusion:**

Collectively, this is the first study from KSA to report potential risk factors of SARS-CoV-2 infection in cancer patients pre- and post-vaccination. Further investigations on these risk factors in a larger cohort are worthwhile to draw a definitive conclusion about their roles in predisposing cancer patients to the infection.

## 1. Introduction

During the COVID-19 pandemic, cancer screening decreased after the implementation of COVID-19 restrictions; and cancer patients faced a significant challenge for risk evaluation [1, 2]. In addition, the risk of SARS-CoV-2 infection in cancer patients has been reported to increase due to the development of an immunosuppressive state, which results from cancer itself and therapeutic processes such as cytotoxic chemotherapy [3–5].

The risk of severe complications and worse outcomes from COVID-19 were found to be associated with cancer in previous studies [6, 7]. Data from earlier reports showed that cancer patients with COVID-19 were more likely to have an increased risk of short-term mortality [8–11]. Similarly, a cohort study found a high rate of in-hospital mortality and one-year all-cause mortality in COVID-19 patients with cancer compared to non-COVID-19 patients with cancer [12]. Furthermore, COVID-19 patients with cancer were found to develop crisis state more than those with no cancer [3]. Age (>65 years) and treatment with immune checkpoint inhibitors (ICIs) of cancer patients were reported to be strong independent predictors for severe respiratory illness due to COVID-19 [13–15]. A cohort study from nine hospitals in China identified factors of COVID-19 severity in cancer patients, including: older age, advanced tumor stage, reduced albumin-globulin ratio, elevated IL-6, reduced CD4+ T cells, procalcitonin, D-dimer, N-terminal pro-B-type natriuretic peptide, reduced lymphocytes and elevated TNF-α [7].

Among Cancer types, a large study of 2152 patients concluded that hematological cancer patients were associated with higher risk of developing COVID-19 versus non-hematological cancer cases [16]. This observation was also supported by other separate reports [17, 18]. Furthermore, a study conducted in Norway involving 547 cancer patients out of total of 8410 patients with SARS-CoV-2 infection concluded that patients with endocrine tumors together with leukemia and lymphomas were at greater risk of developing COVID-19 [19]. Another study reported prostate cancer patients to be the most susceptible group among solid tumor patients to SARS-CoV-2 infection [18].

Immunogenicity following COVID-19 vaccination in cancer patients showed lower levels of SARS-CoV-2 anti-spike IgG antibodies as compared to healthy vaccinated controls after the second dose of the BNT162b2 vaccine [20]. Furthermore, impaired immunogenicity to the BNT162b2 vaccine in patients with solid cancers was reported [21, 22]. Low antibody responses were reported in multiple myeloma patients after the first dose of the BNT162b2 vaccine [23], and chronic lymphocytic leukemia after two doses of the BNT162b2 vaccine [12].

Given the poor clinical outcomes of COVID-19 and the reduced immune response to the vaccination in cancer patients, the characterization of predisposing factors to SARS-CoV-2 infection in this susceptible group is urgently needed to aid in the prevention of COVID-19 and to improve vaccination strategies. While published work from KSA reported comorbidities; such as diabetes, hypertension, cardiovascular diseases, and renal disease to be risk factors for contracting SARS-CoV-2 infection, none of these studies focused on SARS-CoV-2 infection in cancer patients [24–26]. In addition, most of the published work from KSA on risk factors of SARS-CoV-2 infection was reported independently of vaccination against the virus [27, 28]. Therefore, the impact of the reported risk factors in predisposing people to SARS-CoV-2 infection post-vaccination remains to be addressed. To the best of our knowledge, no earlier study from KSA investigated risk factors of SARS-CoV-2 infection in cancer patients before and after vaccination was reported. Therefore, in the present work, we compared clinical and laboratory findings of cancer patients from King Abdulaziz Medical City, Riyadh, KSA who were infected with SARS-CoV-2 pre-vaccination or post-vaccination (1^st^ dose) with that of a control cancer group (no infection pre- or post-vaccination).

## 2. Methods

### 2.1 Collection of clinical and laboratory data

Clinical data and laboratory findings of adult cancer patients were retrospectively retrieved from electronic health records at King Abdulaziz Medical City, Riyadh, KSA during the time period between 1 April 2021 and 31 January 2022 (IRB approval number: RC20/180). The clinical data include (1) patients’ age, gender, nationality and body mass index (BMI); (2) co-morbidities, such as diabetes, chronic kidney disease, hemodialysis, lung disease, hypertension and hyperlipidemia; (3) cancer features including type, stage, Eastern Cooperative Oncology Group (ECOG) performance status, and type of cancer therapy; (4) vaccination data: vaccine type, number of administrated doses and duration between doses. The laboratory findings were hemoglobin concentration (Hb), white blood cells (WBCs) count, lymphocyte count, neutrophil count, neutrophil to lymphocyte ratio (NLR), creatinine concentration, estimated glomerular filtration rate (eGFR). All laboratory parameters were tested prior to infection with SARS-CoV-2 and vaccination against the virus. All lab tests were conducted according to standard procedures of the Clinical Pathology and Laboratory Medicine Department at King Abdulaziz Medical City, which is an accredited lab by the College of American Pathologists (CAP). The study was approved by the IRB (IRB approvals for protocols: RC20/180 and NRC21R-120-03) at King Abdullah International Medical Research Center (KAIMRC). Based on the IRB approvals, consent forms were waived; and clinical and lab data were collected from patients Electronic Medical Records by the Research Data Management department at KAIMRC and delivered to the PI as fully anonymized data. No subject is identifiable in the study dataset.

### 2.2 Inclusion and exclusion criteria

A group of criteria were used to accept or reject data for analysis. The study was conducted only on data from adult cancer patients. Only patients with malignant tumours were included in the study; those with benign tumors were excluded. Of the patients with malignant tumors, only those on active therapy were accepted for analysis. Patients with cancer in a long-term remission were not included in the study. Only patients with known information regarding SARS-CoV-2 vaccination status and SARS-CoV-2 infection (confirmed by Polymerase chain reaction (PCR)) status were accepted for analysis. For a lab test result to be included in the study it has to be conducted prior to SARS-CoV-2 infection and/or SARS-CoV-2 vaccination.

### 2.3 Statistical analysis

Prism Graph Pad software (version 7) was used for the statistical analysis. Unpaired student *t*-test was employed for the calculation of significance (*p* value) of continuous variables. Fisher’s exact test was used for the calculation of significance of categorical variables. Odds ratio and 95% confidence interval (95% CI) were calculated using Baptista-Pike method for categorical variables. Statistically significant findings were those reported with *p* value ≤ 0.05.

## 3. Results

### 3.1 Characteristics of the study cohort

**Fig 1:**
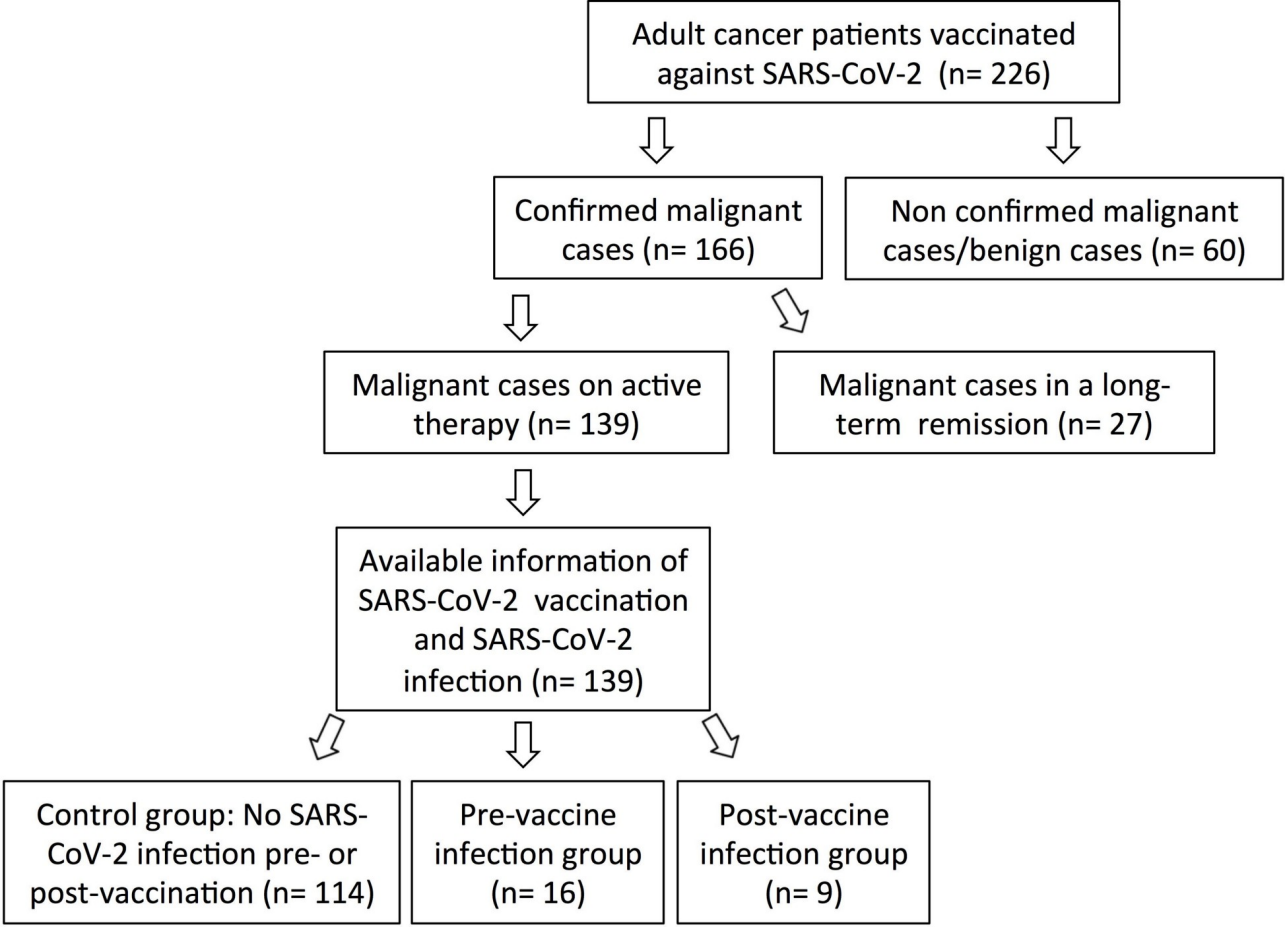
Flowchart of patients from whom data were collected.

The number of cancer patients who were found to meet the inclusion criteria was 139 (Fig 1). The age of the studied patients ranged from 24 to 86 years with median of 54 years. Of the total patients, 91 (65%) were females and 49 (35%) were males. The patients were from 14 different countries; the majority were Saudis (61%) followed by Filipinos (29%). The data showed that the patients were (i) uninfected with SARS-CoV-2 pre-vaccination and remained uninfected post-vaccination (control group; n = 114; 81%), (ii) pre-vaccination infected group (n = 16; 11%), or (iii) post-vaccination infected group (n = 9; 6%). For the latter, all infections occurred after the first dose of vaccine at 57 to 115 days (mean = 80 days). The duration between vaccination (first dose) and the collection of data was 90 to 363 days (mean = 255 days) in all patients. Two vaccines (Oxford-AstraZeneca and Pfizer-BioNTech) were used to vaccinate patients; Oxford-AstraZeneca was the most commonly used (93%; n = 129) for the first dose. For the second dose, Oxford-AstraZeneca and Pfizer-BioNTech were almost equally administrated (47% [n = 65] and 53% [n = 74], respectively). Heterogeneous vaccinations were given to 48% (n = 67) of the control group and 44% (n = 72) of the post-vaccination infected group.

### 3.2 Comorbidity factors

The comorbidities in the three groups of patients were investigated to determine whether they predispose patients to SARS-CoV-2 infection. Comorbidity analysis (Table 1) shows that pre-vaccinated patients receiving hemodialysis and diabetic patients had a significant high risk of infection. The proportion of patients undergoing hemodialysis in the pre-vaccination infected group was 13% (n = 2) as compared to only 1% (n = 1) in the control group (*p* = 0.04; odds ratio = 16.1; 95% CI = 1.7 to 234). Likewise, diabetic patients represented 63% (n = 10) of the pre-vaccination infected group as compared to 35% (n = 40) of the control group (*p* = 0.04; odds ratio = 3.1; 95% CI = 1.1 to 9.2). For the post-vaccination infected patients, no comorbidity factors were found to increase the risk of the infection. Gender and vaccine type did not differ significantly in the infected groups as compared to the control group.

**Table 1:** Comorbidity factors.

Comorbidity	Control (n = 114)	Pre-vaccine infection (n = 16)	*p* value (odds ratio; 95% CI) of pre-vaccine infection VS control	Post-vaccine infection (n = 9)	*p* value (odds ratio; 95% CI) of post-vaccine infection VS control
Hemodialysis	1% n = 1	13% n = 2	0.04 (16.1; 1.7 to 234)	0% n = 0	1 (0; 0 to 114)
Diabetes	35% n = 40	63% n = 10	0.04 (3.1; 1.1 to 9.2)	56% n = 5	0.28 (2.3; 0.6 to 7.7)
Hyperlipidemia	12% n = 14	0% n = 0	0.21 (0; 0 to 1.6)	22% n = 2	0.33 (2; 0.4 to 9)
Chronic Kidney disease	7% n = 8	13% n = 2	0.35 (1.9; 0.4 to 9.6)	0% n = 0	1 (0; 0 to 7)
Obesity	10% n = 11	0% n = 0	0.36 (0; 0 to 2.2)	0% n = 0	1 (0; 0 to 4.5)
Lung disease	11% n = 12	13% n = 2	0.68 (1.2; 0.2 to 5.2)	0% n = 0	0.59 (0; 0 to 4)
Hypertension	39% n = 44	45% n = 7	0.78 (1.2; 0.5 to 3.5)	44% n = 4	0.73 (1.3; 0.4 to 4.6)
Gender	Male 65% n = 74	Male 69% n = 11	1.00 (1.2; 0.4 to 3.2)	Male 56% n = 5	0.72 (0.7; 0.2 to 2.3)
	Female 35% n = 39	Female 31% n = 5		Female 44% n = 4	
Vaccine type	PF 7% n = 7	N/A	N/A	PF 0% n = 0	1 (0; 0 to 6)
	AZ 93% n = 105	N/A		AZ 100% n = 9	

This table compares comorbidity factors of the three groups of patients (control group versus pre-vaccination infected group; and control group versus post-vaccination infected group) to identify risk factors of contracting SARS-CoV-2 infection in cancer patients before or after vaccination. The findings shown are percentage of patients followed by number of patients. CI: confidence interval; AZ: AstraZeneca; PF: Pfizer.

### 3.3 Cancer characteristics

Cancer characteristics of the three groups of patients were investigated (Table 2), and only types of cancer that were reported in two or more patients of any of the groups were studied. Papillary thyroid cancer appeared to be a risk factor of contracting the infection pre-vaccination (*p* = 0.03; odds ratio = 3; 95% CI = 1 to 9) and post-vaccination (*p* = 0.01; odds ratio = 5; 95% CI = 1.3 to 17). The proportion of papillary thyroid cancer patients was 44% (n = 7) of the pre-vaccination infected group and 56% (n = 5) of the post-vaccination infected group as compared to 20% (n = 23) of the control group. On the other hand, breast cancer was significantly less common in the pre-vaccination infected group (6%; n = 1; *p* = 0.05; odds ratio = 0.2; 95% CI = 0 to 1) and in the post-vaccination infected group (0%; n = 0; *p* = 0.06; odds ratio = 0; 95% CI = 0 to 1) as compared to the control group (29%; n = 33). Other included types of cancer did not show significant association with a particular group of patients. Analysis of cancer stage showed that the pre-vaccination infected group was at an earlier stage of cancer as compared to the control group (*p* = 0.01).

**Table 2:** Cancer characteristics.

Type of cancer	Control (n = 114)	Pre-vaccine infection (n = 16)	*p* value (odds ratio; 95% CI) of pre-vaccine infection VS control	Post-vaccine infection (n = 9)	*p* value (odds ratio; 95% CI) of post-vaccine infection VS control
Papillary thyroid cancer	20% n = 23	44% n = 7	0.03 (3; 1 to 9)	56% n = 5	0.01 (5; 1.3 to 17)
Breast cancer	29% n = 33	6% n = 1	0.05 (0.2; 0 to 1)	0% n = 0	0.06 (0; 0 to 1)
Endometrial cancer	5% n = 6	13% n = 2	0.26 (2.6; 0.5 to 12)	0% n = 0	1 (0; 0 to 7.6)
Rectal cancer	2% n = 2	7% n = 1	0.33 (3.7; 0.2 to 33)	0% n = 0	1 (0; 0 to 28)
Renal cell cancer	4% n = 5	0% n = 0	1 (0; 0 to 5)	11% n = 1	0.37 (2.7; 0.2 to 18)
Lung cancer	3% n = 3	0% n = 0	1 (0; 0 to 8)	0% n = 0	1 (0; 0 to 15)
Ovarian cancer	3% n = 3	0% n = 0	1 (0; 0 to 8.)	11% n = 1	0.26 (4.6; 0.3 to 33)
Bladder cancer	4% n = 4	0% n = 0	1 (0; 0 to 8)	11% n = 1	0.32 (3.4; 0.3 to 26)
Colon cancer	4% n = 4	0% n = 0	1 (0; 0 to 8)	0% n = 0	1 (0; 0 to 15)
Laryngeal squamous cell cancer	2% n = 2	0% n = 0	1 (0; 0 to 15)	0% n = 0	1 (0; 0 to 28)
Cancer stage	2.1 (1) n = 55	1.2 (0.63) n = 6	0.01	2.5 (1.3) n = 4	0.57

Cancer characteristics of the three groups of patients (control group versus pre-vaccination infected group; and control group versus post-vaccination infected group) were compared to identify risk factors of contracting SARS-CoV-2 infection in cancer patients before or after vaccination. Type of cancer-related findings shown here are percentage of patients followed by number of patients. For ECOG/PS and cancer stage the findings are shown as follow: average, (standard deviation) and number of patients. CI: confidence interval.

### 3.4 Regimen of cancer therapy

An investigation was conducted to determine association between a regimen of cancer treatment and one of the three patient groups. For simplicity, only treatments that were administered to two or more patients in either of the groups were studied (S1 Table). Combined therapy (combination of two or more types of therapy; chemotherapy, hormonal therapy, radiation therapy and/or surgery) was less common in the pre-vaccination infected group (12% n = 2; *p* = 0.02; odds ratio = 0.2; 95% CI = 0 to 0.8) and in the post-vaccination infected group (11%; n = 1; *p* = 0.04; odds ratio = 0.2; 95% CI = 0 to 1) compared with the control group (42%; n = 48). Nevertheless, radioactive iodine therapy (RAI) alone was more common in the pre-vaccination (31%; n = 6; *p* = 0.05; odds ratio = 3; 95% CI = 1 to 9) and post-vaccination infected groups (56%; n = 5; *p* = 0.01; odds ratio = 6.3; 95% CI = 2 to 21) as compared to the control (17%; n = 19). No association between other forms of treatment and either of the patient groups was found.

### 3.5 Laboratory findings

Laboratory parameters of the three groups were investigated (Table 3). Neutrophil to lymphocyte ratio (NLR) was found to be lower in the post-vaccination infected group (NLR = 1.5) as compared to the control (NLR = 2.5; *p* = 0.01). None of the other lab data differed significantly between the three groups.

**Table 3:** Comparison of laboratory findings.

Parameter	Control: mean (SD) number of patients	Pre-vaccine infection: mean (SD) number of patients	*p* value (pre-vaccine infection VS control)	Post-vaccine infection: mean (SD) number of patients	*p* value (post-vaccine infection VS control)
Lymphocyte count (1000/micL)	2.1 (0.86) n = 62	2.6 (1.5) n = 6	0.38	2.12 (1.2) n = 6	0.86
WBC count (1000/micL)	6.6 (2) n = 62	6.2 (1) n = 6	0.48	6.3 (1) n = 6	0.54
NLR	2.5 (2) n = 62	2 (1.4) n = 6	0.50	1.5 (0.7) n = 6	0.01
Serum creatinine (micM/L)	80 (51) n = 67	73 (26) n = 7	0.70	73 (32) n = 7	0.7
eGFR	89 (26) n = 67	96 (23) n = 7	0.47	94 (38) n = 7	0.76
Neutrophil count (1000/micL)	4.2 (2.4) n = 62	4.1 (2) n = 6	0.88	3.9 (1.8) n = 6	0.76
Hb (mg/dL)	132 (18) n = 62	126 (19) n = 6	0.55	131 (21) n = 6	0.92

Laboratory findings of the three groups of patients (control group versus pre-vaccination infected group; and control group versus post-vaccination infected group) were compared to identify risk factors of contracting SARS-CoV-2 infection in cancer patients before or after vaccination. Data shown are average, (standard deviation) and number of patients. NRL: neutrophil to lymphocyte ratio; WBC: white blood cell; Hb: hemoglobin; eGFR: estimated glomerular filtration rate.

## 4. Discussion

SARS-CoV-2 infection has been shown to be severe when associated with cancer. COVID-19 mortality rate was reported to increase by three folds in cancer patients as compared to the general population [29, 30]. Among cancer patients, those with hematological malignancies appeared to be the most susceptible group with regard to COVID-19 mortality [29, 30]. In addition to the deteriorating prognosis of COVID-19 in cancer patients, SARS-CoV-2 vaccination outcomes in cancer patients are not as satisfactory as in general population. Vaccination in cancer patients resulted in a limited production of SARS-CoV-2 anti-spike antibodies [20–22]. This low immune response indicates that even post-vaccination, cancer patients may remain prone to the infection as compared to vaccinated healthy people. Therefore, characterizing risk factors that predispose cancer patients to COVID-19 is, indeed, required to aid the prevention of the infection in this susceptible group.

In the present work, comorbidity factors, such as hemodialysis and diabetes, were identified as risk factors of the infection in cancer patients prior to vaccination. The findings of diabetes in this study broadly support previous studies that linked diabetes to SARS-CoV-2 infection as a risk factor [31–35]. In the context of Saudi Arabia, studies on adult COVID19 patients reported diabetes to increase the chance of contracting SARS-CoV-2 infection [24, 25]. In addition to diabetes, several reports have shown that patients who are on hemodialysis could have increased susceptibility to COVID-19 before vaccination [36–38], but up to date there is no report found in the literature on COVID-19 susceptibility of hemodialysis cancer patients.

Despite the poor vaccination response in cancer patients, those with breast cancer showed the highest levels of vaccine-induced antibodies compared with patients having other malignancies [39]. Furthermore, in non-vaccinated cancer patients, those with breast cancer were found to be less prone to SARS-CoV-2 infection [40]. In line with these two findings, our data showed that breast cancer is more prevalent in the control group as compared to patients who were infected either pre- or post-vaccination. In contrast to breast cancer, papillary thyroid cancer was found to be a risk factor of the infection in pre-vaccinated and post-vaccinated groups. The published data on thyroid cancer with COVID-19 is scarce; and ambiguity remains on whether association between COVID-19 and thyroid cancer exists [41, 42]. However, thyroid dysfunctions like thyrotoxicosis or low-T3 syndrome have been reported to occur as a result of COVID-19 [43]. RAI therapy, which is commonly prescribed for patients with papillary thyroid cancer, was also associated with the infection pre- and post-vaccination. This finding could be factored by the association of papillary thyroid cancer with COVID-19 shown here. However, cumulative doses of RAI were also reported to induce toxicity in bone marrow causing reduction in the lymphopoiesis among other immune cells [44–47], which may indicate insufficient induction of immune responses.

Increases in neutrophil counts, drop in lymphocyte counts and higher NLR in COVID-19 patients are associated with poor prognosis [48, 49]. To the best of our knowledge, no data on baseline NLR was reported in the context of SARS-CoV-2 infection in cancer patients. Decreased CD8 T lymphocyte counts were shown as a response to inactivated SARS CoV2 vaccine [50]. In multiple myeloma patients, decrease in lymphocyte counts post-vaccination was associated with poor immunogenicity [51]. In the present work, we found NLR measured prior to vaccination to be significantly reduced in patients with infections post-first dose of vaccination as compared to the control group.

Majority of the previously published studies on COVID-19 and cancer investigated risk factors associated with the severity of COVID-19 [3, 6–12, 52]. Few studies attempted to characterize predisposing factors of SARS-CoV-2 infection in cancer patients [16–19]. Of the latter, the majority compared clinical and laboratory data of COVID-19 patients with cancer to that of COVID-19 patients without cancer as a control group. In the present work; however, we compared clinical data and laboratory findings (baseline that were measured prior to the infection or vaccination) in cancer control group without SARS-CoV-2 infection and cancer groups with SARS-CoV-2 infection occurring pre- or post-vaccination. Using cancer patients without COVID-19 as a control group has a better chance to truly identify risk factors that predispose a cancer patient to the infection; and reduce bias that could result from pre-selection of only COVID-19 positive cancer patients from the entire cancer population.

There are some limitations of the present work. The clinical and lab data were retrieved from a single-center, rendering the current work to be a reflection of a single experience. Furthermore, the inclusion and exclusion criteria applied to the present study limited the size of the patient cohort. It should be noticed that the availability of clinical data of Saudi patients with COVID19 that could be used to identify risk factors of SARS-CoV-2 infection in sub-group of patients is limited even for common diseases; such as diabetes (n = 439) [24] and cardiovascular diseases (n = 264) [53]. These two studies were conducted independently of vaccination status, which would decrease the number of patients if it had been used as inclusion criterion. We think that effort should be made in the future to study the identified risk factors in a larger cohort of cancer patients from multiple hospitals to draw a definitive conclusion about their roles in predisposing cancer patients to the infection pre- and post-vaccination.

## 5. Conclusion

Overall, we compared clinical and lab data of cancer patients who were vaccinated against SARS-CoV-2 and acquired SARS-CoV-2 infection either prior to or pre-vaccination with that of a cancer control group with no infection before or after SARS-CoV-2 vaccination. Our work identified a number of risk factors for SARS-CoV-2 infection in cancer patients. Hemodialysis, diabetes, papillary thyroid cancer, treatment with RAI and reduced NLR were found to increase the chance of contracting the infection and having COVID-19 in cancer patients (pre- and/or post-vaccination). In contrast, combined therapy and breast cancer appeared to reduce the chance of SARS-CoV-2 infection (pre- and post-vaccination). This study is the first from KSA to report risk factors of SARS-CoV-2 infection in cancer patients before and after vaccination. These risk factors merit further evaluation in a larger number of patients from multiple hospitals to validate their involvement in contracting the infection in cancer patients.

## Supporting information

S1 Table Comparison of therapy regimen. Data shown are: percentage of patients followed by number of patients. CI: confidence interval.
(DOCX)

S2 Table Minimal data set. This table shows the minimal data set that was used in this study.
(PDF)
