# Supplementary material for: Risk factors of SARS-CoV-2 infection in cancer patients pre- and post-vaccination
Source: PLoS One. 2022 Aug 9;17(8):e0272869. doi: 10.1371/journal.pone.0272869 (PMC9362932; doi:10.1371/journal.pone.0272869)
Supplement: S1 Table — Data shown are: percentage of patients followed by number of patients. CI: confidence interval. (DOCX) [file pone.0272869.s001.docx]

**S1 Table 1: Comparison of therapy Regimen**

| Type of therapy | Control (n=114) | Pre-vaccine infection (n=16) | *p* value (odd ratio; 95% CI) of pre-vaccine infection VS control | Post-vaccine infection (n=9) | *p* value (odd ratio; 95% CI) of post-vaccine infection VS control |
| --- | --- | --- | --- | --- | --- |
| Combined therapy | 42% = 48 | 12% n=2 | 0.02 (0.2; 0 to 0.8) | 11% n=1 | 0.04 (0.2;  0 to 1) |
| Radioactive iodine therapy alone | 17% n=19 | 31% n=6 | 0.05 (3; 1 to 9) | 56% n=5 | 0.01 (6.3;  2 to 21) |
| Surgery alone | 28% n=32 | 44% n=7 | 0.24 (2; 1 to 6) | 22% n=2 | 1 (0.7;  0.1 to 4) |
| Surgery, radiotherapy and hormonal therapy | 2% n=2 | 6% n=1 | 0.33 (3.7; 0.2 to 33) | 0% n=0 | 1 (0; 0 to 28) |
| Granulocyte colony stimulating factor | 7% n=8 | 0% n=0 | 0.59 (0; 0 to 3.5) | 0% n=0 | 1 (0; 0 to 7) |
| Surgery, chemotherapy and hormonal therapy | 2% n=2 | 0% n=0 | 1 (0; 0 to 15) | 0% n=0 | 1 (0; 0 to 28) |
| Chemotherapy and radiotherapy | 2% n=2 | 0% n=0 | 1 (0; 0 to 15) | 0% n=0 | 1 (0; 0 to 28) |
| Surgery and radiotherapy | 3% n=3 | 0% n=0 | 1 (0; 0 to 8) | 0% n=0 | 1 (0; 0 to 15) |
| Surgery, chemotherapy and radiotherapy | 4% n=5 | 0% n=0 | 1 (0; 0 to 8) | 0% n=0 | 1 (0; 0 to 15) |
| Surgery and chemotherapy | 7% n=8 | 0% n=0 | 0.59 (0;  0 to 3.5) | 11% n=1 | 0.5 (1.7; 0.14 to 12) |
| Chemotherapy alone | 5% n=6 | 6% n=1 | 1 (1.2; 0 to 8) | 0% n=0 | 1 (0; 0 to 7) |
| Surgery, chemotherapy and hormonal therapy | 6% n=7 | 0% n=0 | 0.59 (0; 0 to 4) | 0% n=0 | 1 (0; 0 to 6) |
| Surgery and hormonal therapy | 10% n=11 | 6% n=1 | 1 (0.6; 0 to 4) | 0% n=0 | 1 (0; 0 to 4) |

Data shown are: percentage of patients followed by number of patients. CI: confidence interval.
