# Supplementary material for: Risk factors of SARS-CoV-2 infection in cancer patients pre- and post-vaccination
Source: PLoS One. 2022 Aug 9;17(8):e0272869. doi: 10.1371/journal.pone.0272869 (PMC9362932; doi:10.1371/journal.pone.0272869)
Supplement: S2 Table — This table shows the minimal data set that was used in this study. (PDF) [file pone.0272869.s002.pdf]

S2 Table: Minimal data set.

Patient number	NATIONALITY	age	Infection	Vaccine type (1st dose)	Vaccine type (2nd dose)	diabetes	Hypertension	Hyperlipidaemia	Renal diseases	Lung Diseases
1	United Kingdom (British)	46	Yes	Oxford-AstraZeneca	Pfizer-BioNTech					
2	United Kingdom (Britian)	50	NO	Oxford-AstraZeneca	Oxford-AstraZeneca	yes	yes			
3	Syria	41	NO	Oxford-AstraZeneca	Oxford-AstraZeneca	yes				
4	Syria	44	NO	Oxford-AstraZeneca	Pfizer-BioNTech					
5	Sudan	60	NO	Oxford-AstraZeneca	Pfizer-BioNTech					
6	Sudan	60	NO	Oxford-AstraZeneca	Oxford-AstraZeneca	yes				
7	Sudan	63	Yes	Pfizer-BioNTech	Moderna	yes	yes			
8	South Africa	59	NO	Oxford-AstraZeneca	Oxford-AstraZeneca					
9	Saudi Arabia	24	NO	Oxford-AstraZeneca	Pfizer-BioNTech					
10	Saudi Arabia	25	NO	Oxford-AstraZeneca	Oxford-AstraZeneca					
11	Saudi Arabia	25	NO	Oxford-AstraZeneca	Pfizer-BioNTech	yes				
12	Saudi Arabia	26	NO	Oxford-AstraZeneca	Oxford-AstraZeneca					
13	Saudi Arabia	27	NO	Oxford-AstraZeneca	Pfizer-BioNTech					
14	Saudi Arabia	30	NO	Oxford-AstraZeneca	Oxford-AstraZeneca					
15	Saudi Arabia	30	NO	Pfizer-BioNTech	Pfizer-BioNTech					
16	Saudi Arabia	30	Yes	Oxford-AstraZeneca	Oxford-AstraZeneca					
17	Saudi Arabia	33	Yes	Oxford-AstraZeneca	Pfizer-BioNTech					
18	Saudi Arabia	34	NO	Oxford-AstraZeneca	Oxford-AstraZeneca					
19	Saudi Arabia	34	Yes	Oxford-AstraZeneca	Oxford-AstraZeneca					
20	Saudi Arabia	35	NO	Oxford-AstraZeneca	Pfizer-BioNTech					
21	Saudi Arabia	35	NO	Oxford-AstraZeneca	Pfizer-BioNTech		yes			
22	Saudi Arabia	35	NO	Pfizer-BioNTech	Pfizer-BioNTech					
23	Saudi Arabia	37	NO	Pfizer-BioNTech	Pfizer-BioNTech					
24	Saudi Arabia	38	NO	Oxford-AstraZeneca	Pfizer-BioNTech					
25	Saudi Arabia	38	NO	Oxford-AstraZeneca	Oxford-AstraZeneca	yes		yes		
26	Saudi Arabia	38	NO	Oxford-AstraZeneca	Pfizer-BioNTech					
27	Saudi Arabia	39	NO	Oxford-AstraZeneca	Pfizer-BioNTech					
28	Saudi Arabia	39	Yes	Oxford-AstraZeneca	Oxford-AstraZeneca	yes				
29	Saudi Arabia	40	NO	Oxford-AstraZeneca	Oxford-AstraZeneca					
30	Saudi Arabia	40	NO	Oxford-AstraZeneca	Pfizer-BioNTech					
31	Saudi Arabia	41	NO	Oxford-AstraZeneca	Pfizer-BioNTech					
32	Saudi Arabia	42	NO	Pfizer-BioNTech	Pfizer-BioNTech	yes		yes	yes	
33	Saudi Arabia	43	NO	Pfizer-BioNTech	Pfizer-BioNTech					
34	Saudi Arabia	44	NO	Oxford-AstraZeneca	Pfizer-BioNTech	yes	yes			yes
35	Saudi Arabia	46	NO	Oxford-AstraZeneca	Pfizer-BioNTech					
36	Saudi Arabia	47	NO	Oxford-AstraZeneca	Pfizer-BioNTech					
37	Saudi Arabia	47	Yes	Oxford-AstraZeneca	Oxford-AstraZeneca	yes	yes			
38	Saudi Arabia	48	NO	Oxford-AstraZeneca	Pfizer-BioNTech					
39	Saudi Arabia	48	NO	Oxford-AstraZeneca	Pfizer-BioNTech	yes	yes			
40	Saudi Arabia	49	NO	Pfizer-BioNTech	Pfizer-BioNTech	yes	yes			
41	Saudi Arabia	50	Yes	Oxford-AstraZeneca	Pfizer-BioNTech					

42	Saudi Arabia	51	NO	Oxford-AstraZeneca	Pfizer-BioNTech					
43	Saudi Arabia	51	NO	Oxford-AstraZeneca	Pfizer-BioNTech					
44	Saudi Arabia	51	Yes	Oxford-AstraZeneca	Pfizer-BioNTech		yes			
45	Saudi Arabia	53	NO	Oxford-AstraZeneca	Oxford-AstraZeneca	yes	yes			yes
46	Saudi Arabia	54	NO	Oxford-AstraZeneca	Pfizer-BioNTech					
47	Saudi Arabia	54	Yes	Oxford-AstraZeneca	Pfizer-BioNTech					
48	Saudi Arabia	55	NO	Oxford-AstraZeneca	Pfizer-BioNTech					
49	Saudi Arabia	55	NO	Oxford-AstraZeneca	Pfizer-BioNTech			yes		
50	Saudi Arabia	55	Yes	Oxford-AstraZeneca	not determined	yes	yes			yes
51	Saudi Arabia	55	Yes	Oxford-AstraZeneca	Oxford-AstraZeneca		yes			
52	Saudi Arabia	57	NO	Oxford-AstraZeneca	Pfizer-BioNTech	yes	yes			
53	Saudi Arabia	58	NO	Oxford-AstraZeneca	Oxford-AstraZeneca					
54	Saudi Arabia	58	Yes	Oxford-AstraZeneca	Pfizer-BioNTech	yes	yes			
55	Saudi Arabia	58	Yes	Oxford-AstraZeneca	Oxford-AstraZeneca					
56	Saudi Arabia	60	NO	Oxford-AstraZeneca	Pfizer-BioNTech	yes			yes	yes
57	Saudi Arabia	61	NO	Oxford-AstraZeneca	Pfizer-BioNTech	yes	yes		yes	
58	Saudi Arabia	62	NO	Oxford-AstraZeneca	Oxford-AstraZeneca					
59	Saudi Arabia	63	NO	Oxford-AstraZeneca	Oxford-AstraZeneca	yes				yes
60	Saudi Arabia	63	NO	Pfizer-BioNTech	Pfizer-BioNTech	yes				
61	Saudi Arabia	63	NO	Oxford-AstraZeneca	Oxford-AstraZeneca	yes	yes			
62	Saudi Arabia	63	NO	Oxford-AstraZeneca	Pfizer-BioNTech					
63	Saudi Arabia	63	NO	Oxford-AstraZeneca	Oxford-AstraZeneca	yes				
64	Saudi Arabia	64	NO	Pfizer-BioNTech	Pfizer-BioNTech		yes			
65	Saudi Arabia	64	NO	Oxford-AstraZeneca	Oxford-AstraZeneca	yes		yes		
66	Saudi Arabia	64	NO	Oxford-AstraZeneca	Oxford-AstraZeneca	yes				
67	Saudi Arabia	64	Yes	Oxford-AstraZeneca	Pfizer-BioNTech	yes				
68	Saudi Arabia	65	NO	Oxford-AstraZeneca	Oxford-AstraZeneca	yes	yes			
69	Saudi Arabia	65	NO	Oxford-AstraZeneca	Oxford-AstraZeneca	yes	yes		yes	
70	Saudi Arabia	65	NO	Oxford-AstraZeneca	Pfizer-BioNTech	yes	yes			
71	Saudi Arabia	66	NO	Oxford-AstraZeneca	Oxford-AstraZeneca					
72	Saudi Arabia	66	NO	Oxford-AstraZeneca	Oxford-AstraZeneca		yes			yes
73	Saudi Arabia	66	Yes	Oxford-AstraZeneca	Oxford-AstraZeneca	yes			yes	
74	Saudi Arabia	67	Yes	not determined	not determined	yes	yes	yes		
75	Saudi Arabia	68	NO	Oxford-AstraZeneca	Oxford-AstraZeneca	yes	yes			
76	Saudi Arabia	69	NO	Oxford-AstraZeneca	Pfizer-BioNTech	yes	yes			
77	Saudi Arabia	70	NO	Oxford-AstraZeneca	Oxford-AstraZeneca	yes	yes			
78	Saudi Arabia	70	NO	Oxford-AstraZeneca	Pfizer-BioNTech	yes				yes
79	Saudi Arabia	70	Yes	Pfizer-BioNTech	Pfizer-BioNTech	yes	yes			
80	Saudi Arabia	71	Yes	Oxford-AstraZeneca	not determined	yes			yes	
81	Saudi Arabia	73	NO	Oxford-AstraZeneca	Oxford-AstraZeneca	yes	yes			
82	Saudi Arabia	74	NO	Oxford-AstraZeneca	Oxford-AstraZeneca	yes	yes			
83	Saudi Arabia	75	NO	Oxford-AstraZeneca	Oxford-AstraZeneca					

84	Saudi Arabia	76	NO	Oxford-AstraZeneca	Pfizer-BioNTech					yes
85	Saudi Arabia	76	NO	Oxford-AstraZeneca	Oxford-AstraZeneca	yes		yes		
86	Saudi Arabia	77	NO	Oxford-AstraZeneca	Oxford-AstraZeneca					
87	Saudi Arabia	77	NO	Oxford-AstraZeneca	Oxford-AstraZeneca		yes			
88	Saudi Arabia	77	NO	Oxford-AstraZeneca	Oxford-AstraZeneca	yes	yes			yes
89	Saudi Arabia	79	NO	Oxford-AstraZeneca	Oxford-AstraZeneca		yes			
90	Saudi Arabia	79	Yes	Oxford-AstraZeneca	Pfizer-BioNTech	yes	yes			
91	Saudi Arabia	81	NO	Oxford-AstraZeneca	Oxford-AstraZeneca	yes	yes			
92	Saudi Arabia	81	NO	Oxford-AstraZeneca	Oxford-AstraZeneca	yes	yes			
93	Saudi Arabia	82	Yes	Oxford-AstraZeneca	Oxford-AstraZeneca	yes	yes	yes		
94	Saudi Arabia	84	Yes	Oxford-AstraZeneca	Oxford-AstraZeneca	yes	yes			
95	Saudi Arabia	86	NO	Oxford-AstraZeneca	not determined					
96	Philippines	36	NO	Oxford-AstraZeneca	Pfizer-BioNTech					yes
97	Philippines	38	NO	Oxford-AstraZeneca	Pfizer-BioNTech					
98	Philippines	38	Yes	Oxford-AstraZeneca	Pfizer-BioNTech					
99	Philippines	40	NO	Oxford-AstraZeneca	Oxford-AstraZeneca					
100	Philippines	42	NO	Oxford-AstraZeneca	Pfizer-BioNTech	yes		yes		
101	Philippines	42	Yes	Oxford-AstraZeneca	Oxford-AstraZeneca					
102	Philippines	46	Yes	Oxford-AstraZeneca	Pfizer-BioNTech					
103	Philippines	48	NO	Oxford-AstraZeneca	Pfizer-BioNTech	yes				
104	Philippines	48	NO	Oxford-AstraZeneca	Pfizer-BioNTech		yes			
105	Philippines	49	NO	Oxford-AstraZeneca	Pfizer-BioNTech		yes			
106	Philippines	49	NO	Oxford-AstraZeneca	Pfizer-BioNTech					
107	Philippines	49	NO	Oxford-AstraZeneca	Pfizer-BioNTech		yes	yes		
108	Philippines	49	NO	Oxford-AstraZeneca	Pfizer-BioNTech	yes	yes			
109	Philippines	50	NO	Oxford-AstraZeneca	Oxford-AstraZeneca		yes			
110	Philippines	50	NO	Oxford-AstraZeneca	Pfizer-BioNTech	yes	yes		yes	
111	Philippines	50	NO	Oxford-AstraZeneca	Pfizer-BioNTech					
112	Philippines	50	NO	Oxford-AstraZeneca	Oxford-AstraZeneca					
113	Philippines	51	NO	Oxford-AstraZeneca	Pfizer-BioNTech		yes			
114	Philippines	52	NO	Oxford-AstraZeneca	Pfizer-BioNTech		yes			yes
115	Philippines	53	NO	Oxford-AstraZeneca	Pfizer-BioNTech			yes		
116	Philippines	54	NO	Oxford-AstraZeneca	Oxford-AstraZeneca		yes			
117	Philippines	55	NO	Oxford-AstraZeneca	Oxford-AstraZeneca		yes		yes	
118	Philippines	58	NO	Oxford-AstraZeneca	Pfizer-BioNTech			yes		yes
119	Philippines	59	NO	Oxford-AstraZeneca	not determined	yes	yes			
120	Philippines	59	NO	Oxford-AstraZeneca	Oxford-AstraZeneca		yes	yes		
121	Philippines	60	NO	Oxford-AstraZeneca	Oxford-AstraZeneca	yes	yes	yes		
122	Philippines	60	NO	Oxford-AstraZeneca	Oxford-AstraZeneca		yes	yes		
123	Philippines	63	NO	Oxford-AstraZeneca	Oxford-AstraZeneca		yes			
124	Philippines	65	NO	Oxford-AstraZeneca	Oxford-AstraZeneca	yes				
125	Pakistan	52	NO	Oxford-AstraZeneca	Pfizer-BioNTech	yes				

126	Pakistan	52	Yes	Oxford-AstraZeneca	Pfizer-BioNTech	yes	yes			yes
127	Pakistan	58	NO	Oxford-AstraZeneca	Pfizer-BioNTech		yes			
128	Lebanon	44	NO	Oxford-AstraZeneca	Oxford-AstraZeneca					
129	Kuwait	75	NO	not determined	not determined		yes		yes	
130	Jordan	48	NO	Oxford-AstraZeneca	Pfizer-BioNTech		yes			
131	Finland	36	NO	Oxford-AstraZeneca	Pfizer-BioNTech					
132	Eire (Irish Republic)	60	NO	not determined	not determined					
133	Egypt	50	NO	Oxford-AstraZeneca	Pfizer-BioNTech					yes
134	Egypt	61	NO	Oxford-AstraZeneca	Oxford-AstraZeneca	yes				
135	Egypt	63	NO	Oxford-AstraZeneca	Oxford-AstraZeneca		yes	yes		
136	Egypt	69	NO	Oxford-AstraZeneca	Oxford-AstraZeneca					
137	Czech Republic & Slovakia	57	NO	not determined	not determined			yes		
138		55	NO	Pfizer-BioNTech	Pfizer-BioNTech					
139		85	NO	Oxford-AstraZeneca	Oxford-AstraZeneca		yes		yes	

Patient number	Haemodialysis	confirmed cancer	ECOG/PS	GCSF	treatment	stage
1		breast cancer	N/A	no	hormonal	2
2		breast cancer	N/A	yes	chemotherapy + targeted therapy + hormonal	4
3		papillary thyroid cancer	N/A	no	RAI	
4		breast cancer	N/A	no	surgery + hormonal	1
5		bladder cancer	N/A	no	surgery	
6		bladder cancer	N/A	no	surgery	
7		papillary thyroid cancer	N/A	no	surgery	
8		galbladder cancer	N/A	no	surgery + chemotherapy	2
9		renal cell carcinoma	N/A	no	surgery alone	
10		HCC	N/A	no	surgery	
11		pseudopapillary neoplasm of pancreas	N/A	no	surgery	
12		mucoepidermoid carcinoma of salivary gland	N/A	no	surgery	
13		hodgkin lymphoma	N/A	no	chemotherapy	
14		colon cancer	N/A	no	surgery + chemotherapy	2
15		ovarian dysgerminoma	N/A	no	surgery	
16		papillary thyroid cancer	N/A	no	RAI	
17		borderline mucinous ovarian	N/A	no	surgery	1
18		papillary thyroid cancer	0	no	RAI	
19		dermatofibrosarcoma	N/A	no	surgery	
20		breast cancer	0	yes	surgery + chemotherapy + radiotherapy + targeted therapy	3
21		burkitt lymphoma	N/A	no	chemotherapy	
22		papillary thyroid cancer	N/A	no	RAI	
23		papillary thyroid cancer	N/A	no	RAI	
24		endometrial cancer	N/A	no	surgery	1
25		papillary thyroid cancer	N/A	no	RAI	
26		thymoma	N/A	no	surgery	
27		glioma low grade	N/A	no	observation	
28		endometrial cancer	N/A	no	hormonal	1
29		classical seminoma	0	no	surgery + chemotherapy	1
30		papillary thyroid cancer	0	no	RAI	
31		lung cancer	0	no	surgery + chemotherapy + radiotherapy	4
32		papillary thyroid cancer	N/A	no	surgery	
33		renal cell cancer	N/A	no	surgery alone	
34		breast cancer	0	no	surgery + chemotherapy	2
35		papillary thyroid cancer	N/A	no	RAI	
36		papillary thyroid cancer	N/A	no	RAI	
37		papillary thyroid cancer	N/A	no	RAI	
38		testicular cancer - classical seminoma	0	no	surgery + radiotherapy	1
39		RCC	N/A	no	observation	
40		RCC	N/A	no	surgery	
41		papillary thyroid cancer	1	no	RAI	

42		papillary thyroid cancer	N/A	no	surgery	
43		schwannoma (resected)	N/A	no	surgery	
44		breast cancer	0	no	surgery + hormonal	1
45		breast cancer	0	no	surgery + hormonal	2
46		parotid mucoepidermoid cancer	N/A	no	surgery	
47		bladder	N/A	no	surgery	
48		rectal cancer	1	no	surgery + chemotherapy	3
49		papillary thyroid cancer	N/A	no	RAI	
50		rectal cancer	0	no	chemoradiotherapy	2
51		breast cancer	N/A	yes	surgery + chemotherapy + radiotherapy + hormonal	3
52		papillary thyroid cancer	0	no	RAI	
53		SCC of cervix	N/A	no	chemotherapy + radiotherapy	3
54		papillary thyroid cancer	N/A	no	RAI	
55		papillary thyroid cancer	0	no	RAI	
56		ear scc	1	no	surgery	
57		endometrial cancer	N/A	no	surgery	1
58		breast cancer	0	no	surgery + chemotherapy + hormonal	2
59		breast cancer	1	no	surgery + hormonal	1
60		breast cancer	1	no	surgery + chemotherapy + hormonal	2
61		endometrial cancer	N/A	no	surgery	1
62		endometrial cancer	1	no	surgery + chemotherapy + radiotherapy	
63		parapharyngeal hemangiopericytoma	N/A	no	surgery + radiotherapy	
64		breast cancer	0	no	surgery + hormonal	2
65		rectal cancer	0	no	surgery + chemotherapy + radiotherapy	3
66		basal skin cancer	N/A	no	surgery	
67		papillary thyroid cancer	N/A	no	RAI	
68		colon cancer	1	no	surgery	2
69		bladder cancer	N/A	no	surgery	
70		neuroendocrine tumor of pancreas	N/A	no	surgery	
71		breast cancer and DLBCL	N/A	no	chemotherapy	3
72		endometrial cancer	2	no	surgery + chemotherapy + radiotherapy	1
73	yes	granulosa cell tumor	N/A	no	surgery alone	1
74		RCC	N/A	no	observation	
75		GIST + RCC	0	no	surgery alone	
76		papillary thyroid cancer	2	no	RAI	
77		breast cancer	1	no	surgery + chemotherapy + targeted therapy + hormonal	3
78		lung cancer	1	no	chemoradiotherapy	4
79		giant cell tumor of tendon sheath	N/A	no	surgery	
80	yes	endometrial cancer	N/A	no	surgery	1
81		laryngeal squamous cell cancer	N/A	no	surgery + chemoradiotherapy	
82		breast cancer	N/A	no	surgery + radiotherapy + hormonal	2
83		papillary thyroid cancer	1	no	surgery	

84		castleman disease	N/A	no	chemotherapy	
85		urothelial papillary carcinoma	N/A	no	surgery	
86		laryngeal squamous cell cancer	N/A	no	surgery + chemotherapy + radiotherapy	
87		papillary thyroid cancer	1	no	RAI	
88		papillary thyroid cancer	3	no	RAI	
89		colon cancer	1	no	surgery + chemotherapy	3
90		papillary thyroid cancer	N/A	no	RAI	
91		breast cancer	0	no	surgery + hormonal	1
92		colon cancer	1	no	chemo	2
93		prostate cancer	1	no	hormonal	4
94		prostate cancer	N/A	no	surgery + radiation therapy + hormonal	
95		lung cancer	4	no	radiotherapy	
96		papillary thyroid cancer	N/A	no	surgery	
97		breast cancer	0	no	surgery + chemotherapy + hormonal	2
98		thymic cancer	N/A	no	surgery + chemotherapy	3
99		papillary thyroid cancer	N/A	no	RAI	
100		papillary thyroid cancer	N/A	no	surgery	
101		papillary thyroid cancer	N/A	no	surgery	
102		papillary thyroid cancer	N/A	no	RAI	
103		breast cancer	0	no	surgery + hormonal	1
104		phyllodes tumor of breast	N/A	no	surgery	
105		breast cancer	0	no	surgery + hormonal	2
106		breast cancer	0	yes	surgery + chemotherapy + radiotherapy	3
107		endometrial cancer	N/A	no	surgery + radiation therapy	
108		papillary thyroid cancer	N/A	no	RAI	
109		breast cancer	0	yes	surgery + chemotherapy + hormonal	1
110		breast cancer	1	yes	surgery + chemotherapy + hormonal therapy	2
111		breast cancer	0	no	hormonal therapy	2
112		breast cancer	0	no	surgery + chemotherapy + targeted therapy	2
113		breast cancer	1	yes	chemotherapy + surgery + now on hormonal therapy	2
114		breast cancer	1	no	surgery + hormonal	1
115		papillary thyroid cancer	N/A	no	RAI	
116		papillary thyroid cancer	N/A	no	RAI	
117		breast cancer	N/A	no	surgery + hormonal	1
118		NSCLC	0	no	chemotherapy + radiotherapy	4
119		breast cancer	0	no	surgery + chemotherapy + hormonal	1
120		bladder cancer	N/A	no	surgery	
121		breast cancer	1	no	surgery + chemotherapy + hormonal	2
122		breast cancer	1	no	surgery + chemotherapy + hormonal therapy	3
123		breast cancer and rectal cancer	0	no	surgery + hormonal	1
124		papillary thyroid cancer	N/A	no	surgery	
125		lymphoma	0	no	stem cell transplant	3

126		tongue SCC	N/A	no	surgery	
127		ovarian cancer	N/A	yes	surgery + chemotherapy	1
128		papillary thyroid cancer	N/A	no	RAI	
129		prostate cancer	N/A	no	surgery	
130		SCC of skin	0	no	surgery	
131		ovarian tumor	N/A	no	surgery	3
132		breast cancer	0	no	surgery + hormonal therapy	2
133		breast cancer	0	no	surgery + chemotherapy + hormonal therapy	4
134		ampulla of vater cancer	2	yes	surgery + chemotherapy	
135		breast cancer	0	no	surgery + hormonal	2
136		bladder	N/A	no	surgery alone	
137		breast cancer	N/A	no	surgery + hormonal	1
138		breast cancer	0	no	surgery + hormonal + radiotherapy	2
139	yes	papillary thyroid cancer		no	RAI	

This the minimal data set that was used in this study.
